# Analgesic and Anti-Inflammatory Activities of the Methanolic Stem Bark Extract of *Nyctanthes arbor-tristis* Linn.

**DOI:** 10.1155/2013/826295

**Published:** 2013-07-29

**Authors:** Bibhuti Bhusan Kakoti, Paresh Pradhan, Sudarshana Borah, Kabita Mahato, Mritunjay Kumar

**Affiliations:** Department of Pharmaceutical Sciences, Dibrugarh University, Assam 786004, India

## Abstract

Stem bark of *Nyctanthes arbor-tristis* Linn. was extracted in methanol to evaluate their analgesic and anti-inflammatory activities. The analgesic activity was determined on Wistar albino rats by hot plate method, tail flick assay, and tail immersion method using Morphine sulphate as standard drug at a dose of 5 mg/kg of body weight and the results were expressed as mean increase in latency after drug administration ± SEM. The anti-inflammatory activity was assessed by Carrageenan-induced rat paw oedema using diclofenac sodium as standard drug at a dose of 100 mg/kg of body weight and expressed in terms of mean increase in paw volume ± SEM. Stem bark extract was given at a dose of 250 mg/kg and 500 mg/kg of body weight. Both standard drugs and extract were administered orally to the animals. Control received distilled water orally. Results showed that *Nyctanthes arbor-tristis* Linn. had potent analgesic and anti-inflammatory activities.

## 1. Introduction

Ethnobotanical research done in the last few decades has revealed the anti-inflammatory and analgesic properties of plants cited in the traditional literature. Many herbal preparations are being prescribed as anti-inflammatory and analgesic in the traditional literature. The search for new anti-inflammatory and analgesic agents from the huge array of medicinal plant resources is intensifying. This is because such taxa may hold assurance for the discovery of novel therapeutic agents capable of suppressing, reducing, or relieving pain as well as inflammation [1]. Inflammation is a pathophysiological response of mammalian tissues to a variety of hostile agents including infectious organisms, toxic chemical substances, physical injury, or tumor growth leading to local accumulation of plasma fluid and blood cells [2]. Edema formation, leukocyte infiltration, and granuloma formation represent such components of inflammation [3]. There are two main types of anti-inflammatory agents, namely, glucocorticosteroids and nonsteroidal anti-inflammatory drugs (NSAIDs). Due to having adverse side effects, like gastric lesions, caused by NSAIDs and tolerance and dependence induced by opiates, the use of these drugs as analgesic agents has not been successful in all the cases. Therefore, analgesic drugs which lack those effects are being searched all over the world as alternatives to NSAIDs and opiates. Drug research and development (R & D) is comprehensive, expensive, time-consuming, and full of risk. It is estimated that a drug from concept to market would take approximately 12 years and capitalizing out-of-pocket costs to the point of marketing approval at a real discount rate of 11% yields a total preapproval cost estimate of US$ 802 million [4]. On the contrary, many medicines of plant origin had been used since ages without any adverse effects. It is therefore essential that efforts should be made to introduce new medicinal plants to develop more effective and cheaper drugs. Plants represent a large natural source of useful compounds that might serve as lead for the development of novel drugs [5]. The investigation of the efficacy of plant-based drugs used in the traditional medicine has been paid great attention because they are cheap, have little side effects, and according to WHO still about 80% of the world population rely mainly on plant-based drugs [6].

*Nyctanthes arbor-tristis* Linn is a small sacred ornamental tree known across India for its fragrant white flowers [7, 8]. *Nyctanthes arbor-tristis* Linn is commonly known as Night Jasmine [9, 10]. It is native to Southern Asia, stretching across northern Pakistan and Nepal throughout Northern India and Southeast Thailand. In India, it grows in outer Himalayas and is found in tracts of Jammu and Kashmir, Nepal to East of Assam, Bengal, Tripura extended through the Central Region up to Godavari in the South [11]. It is a large shrub growing to 10 m tall, with flaky grey bark [12], stiff whitish hair, young branches [13], and rough leaves [14]. The flowers are fragrant, with a five- to eight-lobed white corolla with an orange red centre; they are produced in clusters of two to seven together, with individual flowers opening at dusk and finishing at dawn [12]. Calyx is 6–8 mm long, narrowly campanulate, hairy outside, glabrous inside truncate or obscurely toothed or lobed, ciliated. Corolla glaborous is more than 13 mm long; tube is 6–8 mm long, orange coloured, about equalling the limbs; lobes are white and unequally obcordate and cuneate [13]. The leaves are opposite, simple, 6–12 cm long, and 2–6.5 cm broad, with an entire margin. The fruit is a flat brown heart shaped to round capsule 2 cm diameter, with two sections each containing a single seed [12]. These are long and broad, obcordate or nearly orbicular, compressed, 2 celled. Seeds are exalbuminous, testa is thick, and outer layer of large transparent cells is heavily vascularized [13]. The flowers are used as stomachic, carminative, astringent to bowel, antibilious, expectorant, hair tonic, in the treatment of piles and various skin diseases [15], and in the treatment of ophthalmic purposes [13]. The bright orange corolla tubes of the flowers contain a coloring substance nyctanthin, which is identical with *ά*-Crocetin (C_20_H_24_O_4_) from Saffron. The corolla tubes were formerly used for dyeing silk, sometimes together with safflower or turmeric [8]. Traditionally, the powdered stem bark is given in rheumatic joint pain, in treatment of malaria and also used as an expectorant [10]. The bark is used for the treatment of snakebite and bronchitis [15, 16]. The stem bark pounded with *Zingiber officinale* and *Piper longum* is boiled in water and the resultant liquid is taken for two days for the treatment of malaria in Orissa. The resulting paste on mixing with arjuna bark is rubbed on the body to treat internal injury and for joint broken bones [13]. The leaves of *Nyctanthes arbor-tristis* Linn. are used extensively in Ayurvedic medicine for the treatment of various diseases such as sciatica, chronic fever, rheumatism, and internal worm infections, and as a laxative, diaphoretic, and diuretic [17]. Leaves are used in cough. Leaf juice is mixed in honey and given thrice daily for the treatment of cough. Paste of leaves is given with honey for the treatment of fever, high blood pressure, and diabetes [18]. Juice of the leaves is used as digestives, antidote to reptile venoms, mild bitter tonic, laxative, diaphoretic, and diuretic. Leaves are also used in the enlargement of spleen [9, 10]. The leaf juice is used to treat loss of appetite, piles, liver disorders, biliary disorders, intestinal worms, chronic fever, obstinate sciatica, rheumatism, and fever with rigors [19]. The extracted juice of leaves acts as a cholagogue, laxative, and mild bitter tonic. It is given with little sugar to children as a remedy for intestinal ailments. In several cases, it has been found to act efficaciously for malaria fever [8]. The decoction of leaves is extensively used by Ayurvedic physicians for the treatment of arthritis, obstinate sciatica, malaria, and intestinal worms and as a tonic, cholagogue, and laxative [20]. The seeds are used as anthelmintics and in alopecia. It is antibilious and an expectorant, and is also useful in bilious fevers [19]. The powdered seeds are used to cure scurfy affections of scalp, piles, and skin diseases [13]. Despite the relatively widespread use of this plant as a remedy against rheumatic pain, very little is known about the scientific basis of such usage. Considering folk claim on *Nyctanthes arbor-tristis,* we decided to investigate the stem barks of this plant for their analgesic, anti-inflammatory activities. The present investigation was undertaken to demonstrate the pharmacological potential of methanolic extracts by using various animal models and thus to explore the plant for their potent anti-inflammatory and antinociceptive effect.

## 2. Materials and Methods

### 2.1. Collection of Plant Material

The stem barks of *Nyctanthes arbor-tristis* were collected in the month of August 2011 from Dibrugarh University campus, Assam and nearby area. The plant was identified by Dr. B. K. Sinha, Scientist-E & H.O.O, Botanical Survey of India, Eastern Regional Centre, Shillong. The identification number of plant is No. BSI/ERC/Tech/Plant identification/2012/494. The stem bark was dried under shade for 15 days, coarsely powdered, and stored under air tight container for further study.

### 2.2. Preparation of Methanolic Extract

200 gms of air dried stem bark of *Nyctanthes arbor-tristis* was grinded in a mechanical grinder to produce coarse powder. The powder was then extracted with 1000 mL of petroleum ether (40–60°C) in a Soxhlet apparatus until the powder becomes completely exhausted. The defatted material was then extracted with 1000 mL of methanol in a Soxhlet apparatus. Resulting methanolic extracts were filtered, concentrated, dried, and stored in a desiccator for use in subsequent experiment.

### 2.3. Phytochemical Screening

The methanolic extract was subjected to preliminary phytochemical test for the detection of major active moiety. The result of different chemical tests on the methanolic stem bark extract of *Nyctanthes arbor-tristis *showed the presence of alkaloids, amino acids, carbohydrate, flavonoids, glycosides, protein, tannins, and phenolic compounds.

### 2.4. Selection and Maintenance of Animals

Healthy adult albino rats (Wistar Strain) of either sex, weighing between 120 and 150 g were obtained from M/S Chakraborty Enterprises, Kolkata, India. The animals were acclimatized under laboratory condition for 2 weeks before the starting of experiments. They were provided with standard diet and water *ad libitum* and maintained under standard conditions of temperature (24 ± 1°C) and humidity (50%) with an alternating 12 h light/dark cycles. The places where the experiments are conducted were kept very hygienic by cleaning with antiseptic solution. The husk for the purpose of keeping as a bed to the animals was cleaned daily before the experiment. All the studies were performed in conformity with the guidance for care and standard experimental animals study ethical protocols.

### 2.5. Acute Toxicity Test

The acute toxicity of *Nyctanthes arbor-tristis *methanolic extract was determined in Wistar albino rats. The study was carried out as per the guidelines set by OECD. Rats were fasted for 16 h and were randomly divided into groups of six rats per group. Graded doses of the extract (250, 500, 1000, and 2000 mg/kg p.o.) were separately administered to the rats in each of the groups by means of oral gavage tube of rats. All the animals were then allowed free access to food and water and observed over a period of 48 hr for signs of acute toxicity. The number of deaths within this period was recorded.

### 2.6. Evaluation of Analgesic Activity

 The animals were divided into four groups of 5 animals each. The control group received distilled water (p.o.), Test Group 1 was given methanolic extract 250 mg/kg (p.o.),Test Group 2 was given methanolic extract 500 mg/kg (p.o.), and Standard Group was given morphine sulphate 5 mg/kg (i.p.).

 [p.o.—per oral, i.p.—intraperitoneal].

#### 2.6.1. Hot Plate Method

The hot plate test was used to measure analgesic activity by the method described by Eddy [21] with minor modifications. Rats were kept on a hot plate having a constant temperature of 55 ± 1°C. The time taken for either paw licking or jumping was recorded. Each rat was separately placed on the hot plate in order to obtain the animal's response to electrical heat-induced pain (licking of the forepaws and eventually jumping out of the plate). Jumping out of the hot plate was taken as an indicator of the animal's response to heat-induced pain. The time taken for each rat to jump out of the plate (i.e., reaction time) was noted and recorded in seconds. Starting before and 15 min after oral administration (p.o.) of vehicle, the test agents methanolic extracts, respectively, at 250 and 500 mg/kg, (p.o.) and intraperitoneal (i.p.) injection of morphine sulfate at 5 mg/kg, the nociceptive response was measured every 15 min interval over a 90-minute period.

#### 2.6.2. Tail-Flick Assay

The Tail flick assay was used to measure analgesic activity by the method described by Amour & Smith, 1941 [22] with minor modifications in the process. Tail flick method was employed to study the antinociceptive activity in albino rats. A radiant heat automatic tail flick analgesiometer was used to measure response latencies. Basal reaction time of animals to radiant heat was recorded by placing the tip (last 1-2 cm) of the tail on radiant heat source. The tail withdrawal from the radiant heat was taken as end point. The cut-off time of 10–12 s was imposed to avoid tail damage by heat. Animal failing to withdraw its tail in 3–5 s was rejected from study. Three to five basal reaction times for each rat at an interval of 5 min were taken to confirm normal behavior of the animal. Control reaction was recorded twice with 15 min intervals between readings. Starting before and 15 min after oral administration (p.o.) of vehicle, the test agents (methanolic extracts resp. at 250 and 500 mg/kg, p.o.) and intraperitoneal (i.p.) injection of morphine sulfate at 5 mg/kg, the nociceptive response was measured every 15 min interval over a 90-minute period.

#### 2.6.3. Tail Immersion Method

The tail immersion assay was assessed according to the method of Luiz et al. [23]. The animals were held in a suitable restrainer with tail extending out. The tail up to 5 cm was then dipped into a pot of water maintained at 55 ± 0.1°C. The time taken for the rat to withdraw the tail in seconds was considered as the reaction time. Starting before and 15 min after oral administration (p.o.) of vehicle, the test agents (methanolic extracts resp., at 250 and 500 mg/kg, p.o.) and intra peritoneal (i.p.) injection of morphine sulfate at 5 mg/kg, the nociceptive response was measured every 15 min interval over a 90-minute period.

### 2.7. Evaluation of Acute Anti-Inflammatory Activity

#### 2.7.1. Grouping and Dosing of Animals

The animals are divided into four groups of 5 animals each. Control Group was given distilled water (p.o.), Test Group 1 was given Methanolic extract 250 mg/kg (p.o.), Test Group 2 was given Methanolic extract 500 mg/kg (p.o.), and Standard Group was given diclofenac sodium 100 mg/kg (p.o.).

#### 2.7.2. Carrageenan-Induced Paw Edema (Acute Model)

Acute inflammation was produced by injecting 1% solution of carrageenan into plantar surface of rat left hind paw at the dose of 0.1 mL per 100 g body weight according to the method of Winter et al., in 1963 [24]. Control group of rats (*n* = 5) received vehicle. Rats in the test groups received different doses of the methanolic extracts (250 and 500 mg/kg, p.o.), respectively. Standard group received Diclofenac (100 mg/kg, p.o.). After 30 minutes carrageenan solution was injected to the animals of all the groups. The paw volume was measured by dipping the foot in the mercury bath up to the anatomical hairline on lateral malleolus and compared with control animals which received only the vehicle. The paw volume was measured using Digital plethysmometer, Model: PLM 01 (Orchid scientific) which works on the principle of mercury displacement method. Measurement was done immediately before, first, second, third, fourth, and fifth hour following carrageenan injection. The oedema inhibitory activity was calculated according to the following formulas:

 Oedema rate *E*% = ((*V*_*t*_ − *V*_0_)/*V*_0_) × 100

 Inhibition rate *I*% = ((*E*_*c*_ − *E*_*t*_)/*E*_*c*_) × 100

*V*_*t*_ = Rate of edema at *t* hour

*V*_0_ = Rate of edema at 0 hour

*E*_*c*_ = Edema rate in control group at *t* hour

*E*_*t*_ = Edema rate in test group at *t* hour.

## 3. Statistical Analysis

Values for analgesic activity were expressed as “mean increase in latency after drug administration ± SEM” in terms of seconds whereas values for anti-inflammatory activity were expressed as “mean increase in paw volume ± SEM.” The significance of difference between means was analyzed by one-way ANOVA followed by Turkey's multiple comparison tests. The difference was considered significant when *P* < 0.05. All statistical procedures were performed according to the method of Alcaraz [25].

## 4. Results and Discussions

### 4.1. Results and Discussions of Analgesic Activity

In studies carried out by Bhadauria et al., on the analgesic activity of ethanolic and aqueous leaves extract of *Nyctanthes arbor-tristis*, it was found from the percentage inhibition index that ethanolic extract was better analgesic than aqueous extract when compared with standard drug aspirin. In this study, the methanolic extract of the stem bark of *Nyctanthes arbor-tristis *shows statistically significant analgesic activity (by all four applied models) compared to control, standard, and MENA_250_. The results of treatment with the extracts of *Nyctanthes arbor-tristis *are comparable with the standard and it showed significant analgesic activity as shown in Tables 1, 2, and 3. 

### 4.2. Results and Discussions of Acute Anti-Inflammatory Activity

In studies carried out by Bhadauria et al., on the ethanolic and aqueous leaves extract of *Nyctanthes arbor-tristis*, it was found that ethanolic extract was better and potent showing significant decrease as compared with that of standard drug indomethacin, but aqueous extract showed a mild decrease in paw volume [26]. In the present study conducted, as compared with that of control, extract and diclofenac sodium significantly reduced the paw edema hours after carrageenan injection. For the control group, swelling increased progressively to a maximum volume of 1.28 ± 0.02 mL at 4 h after the carrageenan injection. Rats pretreated with the methanolic extract of *Nyctanthes arbor-tristis *stem bark showed a significant anti-inflammatory activity and in a dose-dependant manner when compared with standard drug diclofenac sodium. The inhibition of carrageenan-induced inflammation by methanolic extract of *Nyctanthes arbor-tristis *stem bark may be related to inhibitory effect of the compound on prostaglandin synthesis. The results of anti-inflammatory activity are given in the Tables 4, 5, and 6.  Table 4 shows that the paw volume of control group was increased rapidly after carrageenan injection, otherwise in case of rats pre-treated with methanolic extract shows inhibition of paw edema significantly as compared to standard drug.

## 5. Conclusion

In this study, pharmacological evaluation of anti-inflammatory and analgesic activity of methanolic stem bark extract of *Nyctanthes arbor-tristis* was carried out using different experimental models. The methanolic extract of *Nyctanthes arbor-tristis* produced effect similar to nonsteroidal drug Diclofenac sodium and in a dose-dependent manner. Due to nociceptive stimulation, various mediators are produced like prostaglandin, cytokinin, bradykinin, and so forth, producing acute pain and inflammation.

 The methanolic extracts of the stem bark of *Nyctanthes arbor-tristis* prevent the nociceptive component which may be due to the inhibition of the production of prostaglandin and related compounds. Experimental evidence suggests that the extract reduced the rate of edema in carrageenan-induced rat paw edema model. Similarly, it significantly delayed the reaction time of animals to the heat stimulus. So it is concluded from the previous study that the extract has potent analgesic and anti-inflammatory effect. Though this study was the preliminary step towards screening of this plant, it paves the way for further attention and research to identify the active compounds responsible for biological activities. Further studies could be undertaken to elucidate the exact mechanism of action by which extract exert their analgesic and anti-inflammatory effect. Moreover, the phytochemical and pharmacological exploration is required for exploration of other activities with this important plant.

## Figures and Tables

**Table 1:** Hot plate method: effects of methanolic extracts of *Nyctanthes arbor-tristis* stem barks and morphine on pain induced by hot plate method.

Group	Dose (mg/kg)	Reaction time (in sec)				
		0 min	15 min	30 min	60 min	90 min
Control	—	4.05 ± 0.01	3.89 ± 0.16	4.02 ± 0.32	4.11 ± 0.02	3.78 ± 0.15
Standard	5 (i.p)	4.01 ± 0.12 a*	4.92 ± 0.18 a^@^	7.15 ± 0.11 a^@^	8.87 ± 0.12 a^@^	10.41 ± 0.01 a^@^
MENA_250_	250 (p.o)	3.68 ± 0.005 a^#^b^#^	4.25 ± 0.01 a*b^#^	4.98 ± 0.12 a^#^b^@^	5.57 ± 0.17 a^@^b^@^	6.13 ± 0.20 a^@^b^@^
MENA_500_	500 (p.o)	4.03 ± 0.02 a*b*c^#^	4.68 ± 0.05 a^#^b*c*	6.33 ± 0.10 a^@^b*c^@^	7.18 ± 0.005 a^@^b^@^c^@^	8.02 ± 0.12 a^@^b^@^c^@^

Data are expressed as Mean ± SEM, *n* = 6 animals in each group. One-way ANOVA was carried out using Turkey's multiple comparison tests. Comparisons were made between:

a: Control versus Standard, MENA_250_, and MENA_500_.

b: Standard versus MENA_250_ and MENA_500_.

c: MENA_250_ versus MENA_500_.

Symbols represent statistical significance: *P < 0.05, ^#^ *P* < 0.01, ^@^ *P* < 0.001.

**Table 2:** Tail flick assay: time of measurement of latency of tail flick (sec).

Group	Dose (mg/kg)	Reaction time in (sec)				
		0 min	15 min	30 min	60 min	90 min
Control	—	2.74 ± 0.16	2.81 ± 0.12	2.97 ± 0.18	3.01 ± 0.05	2.82 ± 0.12
Standard	5 (i.p)	2.90 ± 0.12 a^@^	4.12 ± 0.18 a^@^	6.59 ± 0.11 a^@^	9.35 ± 0.10 a^@^	9.18 ± 0.18 a^@^
MENA_250_	250 (p.o)	2.80 ± 0.12 a^@^b^@^	2.99 ± 0.20 a^ns^b^@^	3.67 ± 0.17 a*b^@^	5.47 ± 0.20 a^@^b^@^	5.11 ± 0.06 a^@^b^@^
MENA_500_	500 (p.o)	2.85 ± 0.10 a^@^b*c^@^	3.55 ± 0.12 a*b^ns^c^ns^	5.29 ± 0.12 a^@^b^@^c^@^	7.75 ± 0.23 a^@^b^@^c^@^	6.92 ± 0.16 a^@^b^@^c^@^

Data are expressed as Mean ± SEM, *n* = 6 animals in each group. One-way ANOVA was carried out using Turkey's multiple comparison tests. Comparisons were made between:

a: Control versus Standard, MENA_250_, and MENA_500_.

b: Standard versus MENA_250_ and MENA_500_.

C: MENA_250_ versus MENA_500_.

Symbols represent statistical significance: *P < 0.05, ^#^ *P* < 0.01, ^@^ *P* < 0.001, ns: nonsignificant.

**Table 3:** Tail immersion method: effects of methanolic extract of *Nyctanthes arbor-tristis* stem barks and morphine on pain induced tail immersion method.

Group	Dose (mg/kg)	Reaction time in (sec)				
		0 min	15 min	30 min	60 min	90 min
Control	—	3.02 ± 0.12	2.98 ± 0.17	2.85 ± 0.10	3.08 ± 0.14	3.06 ± 0.11
Standard	5 (i.p)	3.06 ± 0.14 a*	5.23 ± 0.04 a^@^	7.58 ± 0.12 a^@^	9.72 ± 0.12 a^@^	9.15 ± 0.13 a^@^
MENA_250_	250 (p.o)	3.04 ± 0.05 a*b*	3.74 ± 0.13 a^@^b^@^	4.26 ± 0.11 a^@^b^@^	5.34 ± 0.18 a^@^b^@^	5.67 ± 0.20 a^@^b^@^
MENA_500_	500 (p.o)	2.98 ± 0.21 a*b*c^ns^	3.89 ± 0.05 a^@^b^@^c*	5.44 ± 0.10 a^@^b^@^c^@^	6.71 ± 0.15 a^@^b^@^c^@^	7.52 ± 0.16 a^@^b^@^c^@^

Data are expressed as Mean ± SEM, *n* = 6 animals in each group. One-way ANOVA was carried out using Turkey's multiple comparison tests. Comparisons were made between:

a: Control versus Standard, MENA_250_, and MENA_500_.

b: Standard versus MENA_250_ and MENA_500_.

c: MENA_250_ versus MENA_500_.

Symbols represent statistical significance: *P < 0.05, ^#^ *P* < 0.01, ^@^ *P* < 0.001, ns: nonsignificant.

**Table 4:** Carrageenan-induced paw edema (acute model): effects of methanolic extracts of *Nyctanthes arbor-tristis* stem bark and diclofenac on carrageenan-induced paw edema in rats.

Group	Dose (mg/kg)	Paw volume increase (in mL)					
		0 hr	1 hr	2 hr	3 hr	4 hr	5 hr
Control	—	0.63 ± 0.01	0.86 ± 0.03	1.12 ± 0.01	1.23 ± 0.005	1.28 ± 0.02	1.20 ± 0.12
Standard	100 (p.o)	0.61 ± 0.12 a*	0.72 ± 0.06 a*	0.80 ± 0.01 a^#^	0.84 ± 0.04 a^@^	0.78 ± 0.01 a^@^	0.73 ± 0.01 a^@^
MENA_250_	250 (p.o)	0.62 ± 0.01 a*b*	0.82 ± 0.10 a*b*	0.94 ± 0.03 a*b*	1.01 ± 0.01 a^@^b^@^	0.97 ± 0.06 a^@^b^#^	0.92 ± 0.02 a*b*
MENA_500_	500 (p.o)	0.61 ± 0.02 a*b*c^ns^	0.78 ± 0.01 a*b*c*	0.90 ± .12 a*b*c*	0.95 ± 0.01 a^@^b^#^c*	0.89 ± 0.05 a^@^b*c*	0.82 ± 0.01 a^#^b*c*

Data are expressed as Mean ± SEM, *n* = 6 animals in each group. One-way ANOVA was carried out using Turkey's multiple comparison tests. Comparisons were made between:

a: Control versus Standard, MENA_250_, and MENA_500_.

b: Standard versus MENA_250_ and MENA_500_.

c: MENA_250_ versus MENA_500_.

Symbols represent statistical significance: *P < 0.05, ^#^ *P* < 0.01, ^@^ *P* < 0.001.

**Table 5:** Rate of edema formation.

Group	Dose (mg/kg)	Increase in edema (%)					
		0 hr	1 hr	2 hr	3 hr	4 hr	5 hr
Control	—	—	36.25 ± 0.11	77.32 ± 0.14	96.43 ± 0.43	103.14 ± 0.13	100.41 ± 0.23
Standard	100 (p.o)	—	18.34 ± 0.34 a^#^	31.12 ± 0.16 a^#^	37.14 ± 0.41 a^#^	27.34 ± 0.45 a^#^	19.32 ± 0.34 a^#^
MENA_250_	250 (p.o)	—	25.12 ± 0.16 a^#^b^@^	51.26 ± 0.13 a^#^b^@^	62.32 ± 0.32 a^#^b^@^	56.57 ± 0.22 a^#^b^@^	48.43 ± 0.21 a^#^b^@^
MENA_500_	500 (p.o)	—	27.45 ± 0.17 a^#^b^@^c^@^	47.32 ± 0.14 a^#^b^@^c^@^	55.31 ± 0.43 a^#^b^@^c^@^	45.42 ± 0.20 a^#^b^@^c^@^	34.21 ± 0.24 a^#^b^@^c^@^

Data are expressed as Mean ± SEM, *n* = 6 animals in each group. One-way ANOVA was carried out using Turkey's multiple comparison tests. Comparisons were made between:

a: Control versus Standard, MENA_250_, and MENA_500_.

b: Standard versus MENA_250_ and MENA_500_.

c: MENA_250_ versus MENA_500_.

Symbols represent statistical significance: *P < 0.05, ^#^ *P* < 0.01, ^@^ *P* < 0.001, ns: nonsignificant.

**Table 6:** Rate of inhibition of edema (%).

Group	Dose (mg/kg)	Inhibition of edema (%)					
		0 hr	1 hr	2 hr	3 hr	4 hr	5 hr
Control	—	—	—	—	—	—	—
Standard	100 (p.o)	—	50.45 ± 0.12	59.56 ± 0.45	61.34 ± 0.83	73.43 ± 0.21	81.31 ± 0.32
MENA_250_	250 (p.o)	—	11.34 ± 0.16 b^#^	33.45 ± 0.41 b^#^	36.67 ± 0.34 b^#^	45.73 ± 0.32 b^#^	52.13 ± 0.32 b^#^
MENA_500_	500 (p.o)	—	25.34 ± 0.43 b^#^c^@^	39.87 ± 0.65 b^#^c^@^	42.34 ± 0.23 b^#^c^@^	56.67 ± 0.12 b^#^c^@^	66.32 ± 0.14 b^#^c^@^

Data are expressed as Mean ± SEM, *n* = 6 animals in each group. One-way ANOVA was carried out using Turkey's multiple comparison tests. Comparisons were made between:

b: Standard versus MENA_250_, and MENA_500_.

c: MENA_250_ versus MENA_500_.

Symbols represent statistical significance: ^#^ *P* < 0.01, ^@^ *P* < 0.001.
